# *Xenopus* retinal ganglion cell axon extension is unaffected by 5-HT _1B/D_ receptor activation during visual system development

**DOI:** 10.17912/micropub.biology.001076

**Published:** 2023-12-04

**Authors:** Petros Basakis, Aalim Khaderi, Barbara Lom

**Affiliations:** 1 Biology & Neuroscience, Davidson College, Davidson, North Carolina, United States; 2 Systems Biology, Harvard Medical School, Harvard University, Cambridge, Massachusetts, United States

## Abstract

Activating 5-HT _1B/D_ receptors with the agonist Zolmitriptan was previously shown to facilitate *Xenopus* retinal ganglion cell (RGC) axon extension from ectopic eye primordia transplanted to the ventral fin. To determine if 5-HT _1B/D_ receptor activation influenced entopic RGC axonal outgrowth toward the optic tectum during typical visual system development, we reared embryos in 50 μΜ Zolmitriptan then visualized optic tracts with anterograde HRP labeling. Zolmitriptan did not significantly alter entopic RGC extension in the contralateral brain. Consequently, RGC axon extension in ectopic but not entopic locations is influenced by altering serotonergic signaling .

**Figure 1. Entopic RGC axon extension and fasciculation is unaffected by 5-HT 1B/D activation in the developing visual system:**
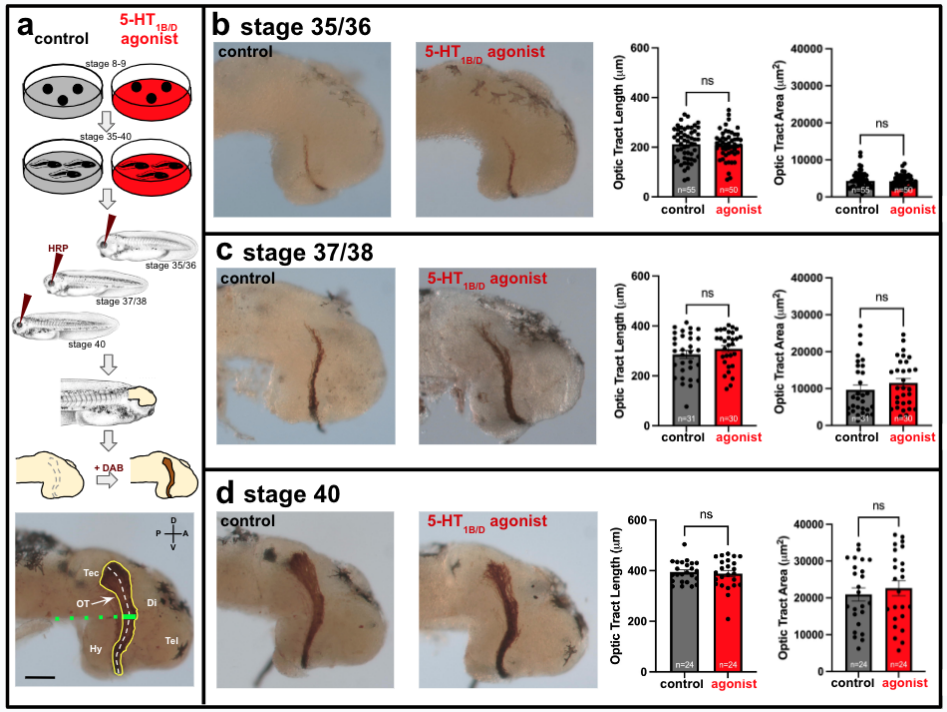
*Xenopus* embryos were reared in 50 μM of the 5-HT _1B/D_ agonist Zolmitriptan or a vehicle control solution beginning just prior to gastrulation (stage 8-9) through stages 35/36, 37/38, or 40 when RGCs are actively navigating along the optic tract (OT) to reach their target in the contralateral optic tectum (Tec; a). To label OTs, the enzyme HRP was introduced into the left eye for anterograde transport by RGC axons. The embryos were fixed and right OTs were then visualized by HRP catalysis of a chromogenic substrate (DAB) in isolated brains. To evaluate axon extension, OT lengths were measured on the lateral surface of the right brain as the distance from the most ventral entry point to the most posterior termination point (dashed white line in a). To evaluate RGC axon fasciculation, both the OT area (yellow outline in a) and OT width (wide solid green line in a) aligned with the ventral brain border (dotted green line in a) were measured. Enhanced serotonergic signaling with the 5-HT _1B/D_ agonist Zolmitriptan did not significantly alter the lengths, areas, or widths of OTs compared to the controls during early (stage 35/36; b), middle (stage 37/38; c), or late (stage 40; d) stages of RGC extension. A, anterior; P, posterior; D, dorsal; V, ventral; Di, diencephalon; Hy, hypothalamus; OT, optic tract; Tec, tectum; Tel, telencephalon. Tadpole illustrations © 2021 Natalya Zahn (www.xenbase.org RRID:SCR_003280; Zahn et al. 2022; CC BY-NC 4.0). Bar = 100 μm; ns at p>0.05; error bars = SEM.

## Description

Retinal ganglion cell (RGC) axons form the optic nerves (ONs) that transmit visual information from each retina to the brain. Improper ON development and/or damage in these afferent pathways can compromise vision. Although injured mammalian ONs typically cannot regenerate, substantially more ON regenerative capacity and restoration of vision is observed in other vertebrate visual systems including zebrafish and *Xenopus* (Gaze 1959; Wilson et al. 1992; Zhao and Szaro 1994; Tanaka and Reddien 2011; Liu et al. 2012; Zou et al. 2013; Whitworth et al. 2017; Kha et al. 2019) . The molecular mechanisms underlying the differential regenerative potentials of optic nerves capable and incapable of regenerating are not fully understood but of considerable interest for developing potential regenerative therapies (Yin et al. 2019; Fague et al. 2021; Miller and Tsai 2023). Consequently, investigating factors that facilitate proper RGC axon development may lead to potential therapies to encourage mammalian ON regeneration in situations of disease or damage.

In the developing *Xenopus* visual system, the first RGCs axons arise by stage 28 with the first active growth cones exiting the retina at the optic nerve head to traverse the optic chiasm, and enter the contralateral brain by stage 32 (Grant and Rubin 1980; Holt 1989) . RGC axons extend along the contralateral brain in a ventral to dorsal fashion making a characteristic caudal turn toward their target, the optic tectum. The first RGC axons reach the optic tectum at stage 37/38 where they initiate synaptogenesis with tectal neurons. By stage 40, many more RGC axons have terminated in the optic tectum and behavioral responses to visual cues are observed by stage 44-45 (Grant and Rubin 1980; Chien and Harris 1994; Dong et al. 2009; McFarlane and Lom 2012; Viczian and Zuber 2014) .

Throughout the central nervous system, serotonin (5-hydroxytryptamine, 5-HT) transmits many important signals from pre- to postsynaptic neurons through seven classes of 5-HT receptors (Pourhamzeh et al. 2022) . Even before synaptogenesis, neurotransmitters play a variety of important roles in neuronal development influencing cell proliferation, motility, axon extension, survival, and differentiation (Haydon et al. 1984; Severin and Kondratyev 1988; Sikich et al. 1990; Lauder 1993; Liu and Lauder 1993; Lima et al. 1994, 1996; Upton et al. 1999; Koert et al. 2001; Fricker et al. 2005; Homma et al. 2006; Bonnin et al. 2007; Xu et al. 2010) . Serotonergic signaling can be dysregulated in a variety of conditions such as addiction, depression, migraine, anxiety, and pain perception that are treated with pharmaceuticals targeting 5-HT _1_ receptors (Gingrich and Hen 2001; Gross et al. 2002; Pytliak et al. 2011; Pourhamzeh et al. 2022) . Alterations in serotonin signaling also influence the regeneration of damaged axons (Sobrido-Cameán et al. 2018; Bajjig et al. 2022). Serotonin’s regulatory influences on neuronal development can be excitatory or inhibitory, varying between and within species depending on ligand-receptor interactions, second messenger systems, the presence of glial cells, and/or the developmental stage (Haydon et al. 1984; Severin and Kondratyev 1988; Sikich et al. 1990; Goldberg et al. 1991; Liu and Lauder 1991; Lauder 1993; Lima et al. 1994; Fricker et al. 2005; Homma et al. 2006; Bonnin et al. 2007; Xu et al. 2010) .

In the mature vertebrate retina, 5-HT is synthesized by amacrine cells that synapse on RGC dendrites in the inner plexiform layer (Vaney 1986; Wässle and Chun 1988). In the *Xenopus* visual system, 5-HT signaling was identified as a potential candidate for facilitating RGC axon extension from donor eyes grafted to ectopic locations distant from the brain (Blackiston et al. 2015, 2017) . RGCs from donor eyes heterotopically transplanted to the ipsilateral flank of host tadpoles could in some cases extend axons that conferred rudimentary vision as detected in a light-mediated learning assay (Blackiston and Levin 2013, 2017) . Specifically, RGC axon extension from ectopic eyes was enhanced by rearing developing embryos in bath applications of either 50 μM serotonin directly (Blackiston et al. 2015) or 50 μM Zolmitriptan, an agonist of 5-HT _1B_ and 5-HT _1D_ receptors (Blackiston et al. 2017) .

This study aimed to determine if similar activation of 5-HT _1B/D_ receptors with 50 μM Zolmitriptan influenced the development of entopic optic nerves as they initially grew from the retina toward their synaptic targets in the contralateral optic tectum. Investigating mechanisms of RGC innervation within the native CNS is more applicable to regenerative situations because ON regeneration within the native visual system is a more likely clinical situation than ectopic retinal transplantation. RGC axon growth *in vivo* was visualized at discrete stages of development using anterograde horseradish peroxidase (HRP) labeling (Cornel and Holt 1992; Chien et al. 1993) . To determine if 5-HT _1B/D_ receptor activation affected entopic RGC initial innervation, tadpoles were reared in 0 μM (vehicle control) and 50 μM Zolmitriptan treatments ( Fig. 1a ). To document the RGC axonal extension in the contralateral brain, optic tract (OT) lengths were measured ( Fig. 1a ) at stage 35/36 when some RGC actively extending in the contralateral brain have initiated a characteristic caudal turn toward the tectum ( Fig. 1b ), at stage 37/38 when the first axons have reached the tectum ( Fig. 1c ), and at stage 40 when many RGCs have ceased extension to terminate at the optic tectum and began initiating synaptogenesis ( Fig. 1d ). We observed that enhanced serotonergic signaling by 50 μM Zolmitriptan did not significantly alter (p>0.05) entopic optic tract lengths at stage 35/36 (-1% average change; Fig. 1b ), stage 37/38 (+8% average change; Fig. 1c ), or stage 40 (+2% average change; Fig. 1d ). To determine if 5-HT _1B/D_ receptor activation affected entopic RGC axon fasciculation, OT areas were measured at stages 35/36 ( Fig. 1b ), 37/38 ( Fig. 1c ), and 40 ( Fig. 1d ), also observing no significant changes between the vehicle control and 50 μM Zolmitriptan conditions (p>0.05). In addition, average OT widths did not significantly differ between control and Zolmitriptan conditions at stages 35/56 (20.1 + 1.6 vs. 18.5 + 1.7 μm), 37/38 (26.0 + 2.2 vs. 31.9 + 2.8 μm), and 40 (44.8 + 4.2 vs. 42.5 + 3.5 μm) suggesting that Zolmitriptan did not modify RGC fasciculation. Consequently, these results suggest that enhanced 5-HT _1B/D_ receptor signaling does not influence entopic *Xenopus* RGC axon bundling or extension toward their tectal target during development.

A prior study examining *Xenopus* RGC axon extension from entopic retinal transplants observed that 50 μΜ Zolmitriptan treatment did not alter donor RGC axonal innervation of the host tectum, yet Zolmitriptan treatment did enhance regeneration of some ectopic retinal transplants to the ipsilateral ventral fin to permit detectable visual learning behaviors in some tadpoles without entopic eyes (Blackiston and Levin 2013; Blackiston et al. 2017) . Although transplanted entopic RGC axons were treated with 50 μM Zolmitriptan for a longer duration (stage 23-47; Blackiston et al. 2017) than in this study of native (non transplanted) entopic RGC axons (stage 8-40), RGC axon outgrowth was not affected by 50 μm Zolmitriptan in either experimental situation in which anatomically typical optic nerves developed. Morphologies of RGC axons extending from entopic retinas differed considerably from those extending from ectopic retinas. Specifically, entopic RGCs fasciculate to form bundled optic nerves ( Fig. 1b -d; Blackiston et al. 2015, 2017) in comparison to RGCs extending from ectopic RGCs that form far more diffuse axonal networks (Blackiston and Levin 2013, Blackiston et al. 2015, 2017) . This difference in RGC extension morphologies may be due to the difference in availability and patterning of guidance cues between the entopic and ectopic locations. Interestingly, the differential effects of 5-HT _1B/D_ receptor activation on RGCs extending toward the brain from entopic (intact and transplanted) and ectopic locations suggest that the nature of the environment through which the RGCs navigated may confer differential sensitivity to enhanced serotonergic signaling. In support of this hypothesis, ectopic RGC axon extension was influenced by pharmaceutical depolarization of host but not donor tissues (Blackiston et al. 2015) .

Expanding this line of investigation to examine potential roles of additional serotonin receptor subtypes in RGC axon extension may be worthwhile because 5-HT _1A_ and 5-HT _2A_ receptors have been implicated in neurite outgrowth (Rojas et al. 2014; Ishima et al. 2015) , 5-HT _1A_ , 5-HT _2B_ and 5-HT _2C_ receptors are expressed in the developing *Xenopus* retina (Marracci et al. 1997; De Lucchini et al. 2005) , and 5-HT _6_ receptors influence dendritic morphogenesis and outgrowth (Lesiak et al. 2018; Pujol et al. 2020) . Moreover, *Xenopus* amacrine cells begin to synthesize 5-HT at approximately stage 35/36 (Ori et al. 2013) and 5-HT _1A_ receptor expression is detected in the eye by stage 41 (Marracci et al. 1997) . Consequently, alterations in serotonergic signaling may not be detectable by RGCs just beginning to express 5-HT receptors during initial RGC axon extension, but detectable by more mature RGCs. Rearing *Xenopus* embryos in solutions with pharmaceutical agents is a convenient experimental strategy that can lead to questions of drug penetration and tissue access. Future experiments could therefore apply pharmacological reagents more directly to the extending RGC axons and the environment through which they navigate via an *in vivo* exposed-brain preparation (Chien et al. 1993; McFarlane et al. 1995; Chien and Harris 1996; Walz et al. 1997) and/or *in vitro* analyses of RGC extension and growth cone morphologies out from retinal neurons growing on defined cellular or acellular surfaces (Gooday 1990; de la Torre et al. 1997; Ruchhoeft et al. 1997; Lom et al. 1998; Campbell et al. 2001; Shewan et al. 2002) . Additionally, *in vivo* extension trajectories, growth cone dynamics, and/or fasciculation of individual RGC axons in the developing optic tract could be examined through techniques that label a small subset of RGC axons *in vivo* (Chien et al. 1993; Lom et al. 1998; Watson et al. 2012; Ruthazer et al. 2013a,b; Erdogan et al. 2016; Jin et al. 2018; Santos et al. 2020) . Elucidating potential contributions of serotonergic signaling to RGC axon extension and pathfinding will enhance understanding of both developmental mechanisms and potential regenerative strategies for afferent retinal neurons.

## Methods

Adult *Xenopus laevis* frogs were maintained in environments following Davidson College IACUC guidelines. Embryos were obtained by overnight paired breedings. Superovulation was encouraged by human chorionic gonadotropin (hCG) hormone treatment (Sive et al. 2000) . Fertilized eggs were dejellied in 2% (w/v) cysteine in 20% Steinberg’s solution and sorted manually to select morphologically appropriately developing early embryos staged according to Nieuwkoop and Faber (1994) and Zahn et al. (2017, 2022). A 50 mM stock solution of the 5-HT _1B/D_ agonist Zolmitriptan was prepared in DMSO with aliquots frozen at -20 °C then thawed immediately before being diluted to 50 μM in 20% Steinberg’s solution (per Blackiston et al 2017). Control embryos were reared in a vehicle control solution of an equivalent concentration of DMSO (1 μl/ml). For each experiment healthy, typically developing pre-gastrula embryos (stage 8-9) were reared in Zolmitriptan or control solutions at temperatures of 15-25 °C at densities of no more than 15 embryos per 10 ml in 60 x 15 mm Petri dishes. Optic nerves at stages 35/36, 37/38, and 40 were anterogradely labeled with horseradish peroxidase (HRP) in L-⍺-lysolecithin as described in Chien et al. (1993). Tadpoles were anesthetized in 0.05% Tricaine-S in 20% Steinberg’s solution. The lens of the left eye was removed and replaced with a similarly sized bolus of HRP in 1% L-⍺-lysolecithin. After 25 minutes for uptake and transport of HRP, the anesthetized embryos were fixed by immersion in 4% glutaraldehyde for one hour. The brains were manually isolated then rinsed three times in PBST (phosphate buffered saline (PBS) with 1% Triton X-100). A solution containing the HRP substrate diaminobenzidine (DAB; 0.7 mg/ml) with hydrogen peroxide (1.6 mg/ml) in 0.6 M TRIS buffer was applied for approximately one minute, followed by three PBST rinses and three PBS rinses. Brains were stored in PBS in a humid chamber at 4 °C prior to imaging the contralateral optic tracts. Digital images of the right lateral view of each labeled brain were collected with TCapture software on a Nikon SMZ1270 stereomicroscope. The lengths of each OT from the ventral entry point to termination point in the optic tectum were measured to quantify RGC axon extension ( Fig. 1a ; dotted white line). Both the area of each OT ( Fig. 1a ; solid yellow outline) and the width of the OT at a diencephalic location ( Fig. 1a ; solid green horizontal line) aligned horizontally to the ventral border of the posterior brain ( Fig. 1a ; green dotted line) were measured. In cases where the most dorsal extent of the OT had not yet crossed a plane horizontal to the ventral brain border, then the width of the most dorsal position of the OT was measured. The experimenter was unaware of experimental conditions during all measurements. Unpaired t-tests compared RGC extension lengths between treatment and control conditions.

## Reagents

**Reagent - Source, Catalog Number, and/or Identifier**

chorionic gonadotropin (hCG) - Intervet Chorulon and Sigma Aldrich CG10

L-cysteine - Sigma Aldrich C7880

3,3'-diaminobenzidine (DAB) - Sigma D4168

glutaraldehyde - Fisher Scientific 02957-1

horseradish peroxidase (HRP) - Sigma P6742

L-α-lysolecihin - Sigma 62962

software (graphing and statistics) - GraphPad Prism 9.4.1

software (image analysis) - ImageJ 1.53t and NIS-Elements

20% Steinberg's solution - recipe per Lom and Cohen-Cory (1999)

tricaine methanesulfonate - Western Chemical Tricaine-S

Triton X-100 - Fisher Scientific LP151

Zolmitriptan - Millipore Sima SML0248
